# Utilizing offspring genotype-by-proxy Mendelian randomization to investigate the causal effect of offspring perinatal traits on maternal health

**DOI:** 10.1093/ije/dyag030

**Published:** 2026-03-09

**Authors:** Alesha A Hatton, Caroline Brito Nunes, Deborah A Lawlor, David M Evans

**Affiliations:** Institute for Molecular Bioscience, The University of Queensland, St Lucia, QLD, Australia; Institute for Molecular Bioscience, The University of Queensland, St Lucia, QLD, Australia; MRC Integrative Epidemiology Unit, University of Bristol, Bristol, United Kingdom; Population Health Science, Bristol Medical School, University of Bristol, Bristol, United Kingdom; Institute for Molecular Bioscience, The University of Queensland, St Lucia, QLD, Australia; MRC Integrative Epidemiology Unit, University of Bristol, Bristol, United Kingdom; Frazer Institute, The University of Queensland, Woolloongabba, QLD, Australia

**Keywords:** Mendelian randomization, maternal health, intergenerational causal inference, perinatal traits, fetal effects, offspring genetic effects

## Abstract

**Background:**

During the perinatal period, the fetus can exert profound effects on processes that alter pre- and postnatal maternal physiology. It is possible to investigate the causal effect of offspring perinatal exposures on their mother’s health using Mendelian randomization (MR). However, analyses need to be adjusted for maternal genotype to avoid confounding. Such analyses are difficult to perform at scale because of the paucity of cohorts across the world with large numbers of genotyped maternal–offspring dyads and parent–offspring trios.

**Methods:**

We introduce the “offspring genotype-by-proxy” MR framework which can be employed in the absence of offspring genetic information to complement existing approaches in the triangulation of causal inference. The basic idea is to use paternal genotypes to proxy the direct effect of their offspring’s genotype on their offspring’s own exposures.

**Results:**

We compare our framework to other MR designs and investigate the consequences of model misspecification and spousal misclassification on statistical power, consistency, and bias. In addition, we discuss the key MR assumptions that prevent these approaches from being appropriate for investigating the effect of many offspring postnatal and later life exposures on maternal health.

**Conclusion:**

Given the increasing availability of datasets such as the UK Biobank that (incidentally) include tens of thousands of genome-wide genotyped spousal pairs and large population biobanks with linked health record data for first-degree relatives, the offspring genotype-by-proxy MR approach could augment causal analyses of offspring perinatal exposures on their mother’s outcomes as implementation is not restricted to datasets with mother–offspring genotype information.

Key Messages

- We developed a new Mendelian randomization (MR) method that enables the investigation of the causal effect of perinatal offspring traits on their mother’s health in the absence of offspring genotype.
- While less powered than MR using similar-sized samples of genotyped mother–offspring pairs, our framework includes an inbuilt control for violation of core assumptions through the inclusion and comparison of causal effect estimates in parous and non-parous spousal pairs.
- Given the increasing availability of datasets such as the UK Biobank that (incidentally) include tens of thousands of genome-wide genotyped spousal pairs, this method provides a complementary approach in the triangulation of causal inference.

## Background

There is a complex interplay between maternal and fetal physiological influences during the perinatal period [1], with offspring perinatal traits plausibly influencing both perinatal and postnatal maternal health. For example, fetal drive, that is the genetic and/or environmental influences originating from the fetus, has been hypothesized to modify maternal physiology as a potential mechanism to increase fetal nutrient delivery and optimize growth [2, 3]. This is evidenced by fetal genetic variants associated with increased maternal glucose concentrations [4], risk of gestational hypertension [5], preeclampsia [6], and hyperemesis gravidarum [7]. Further, fetal genetic predisposition to a higher placental weight increases the risk of maternal preeclampsia and decreases gestational duration [8]. Male fetal sex has also been associated with maternal increased fasting insulin sensitivity and secretion [9] and increased risk of pre-eclampsia and gestational diabetes [10].

**Figure 1:**
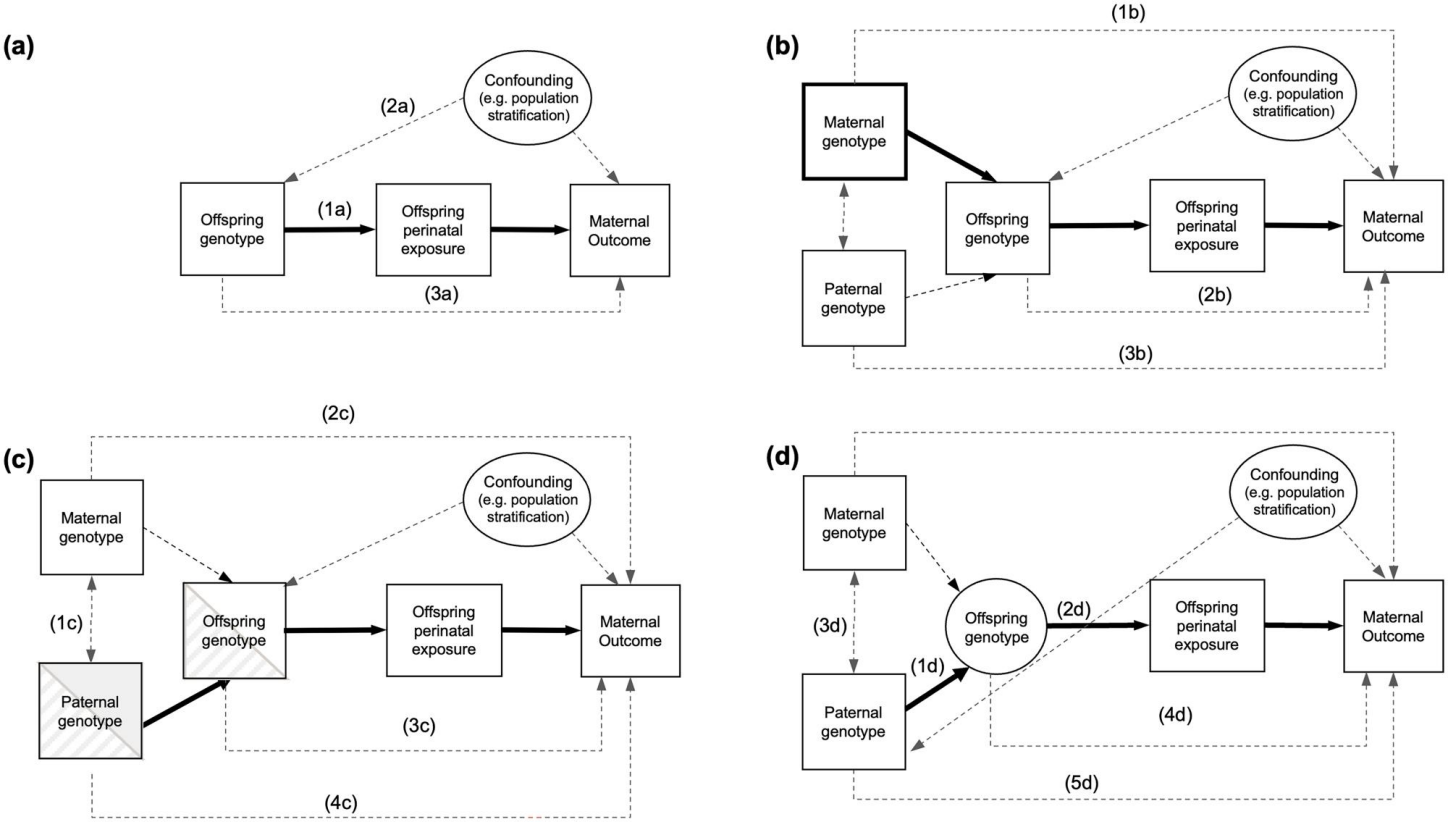
Causal diagram illustrating Mendelian randomization (MR) in the context of investigating the causal effect of an offspring perinatal exposure on a maternal health-related outcome. Observed variables are represented by squares, while latent unobserved variables are represented by circles. Solid arrows indicate the paths of interest in MR. Dotted arrows indicate secondary paths or assumptions. Panel (a) illustrates the MR framework and its core assumptions of relevance (1a), independence (2a), and exclusion restriction (3a). Panel (b) illustrates how maternal genetic variants (that are correlated with the offspring genotype) may violate the independence assumption (1b). However, conditioning on maternal variants (as indicated by the bold box around maternal genotype) blocks path (1b). Path 2b illustrates how offspring genetic variants may violate the exclusion restriction assumption and how paternal genetic variants are a potential source of genetic confounding (3b). Panel (c) illustrates how utilizing paternally transmitted alleles (which should be uncorrelated with maternal genotype in the absence of assortative mating; 1c) should protect MR analysis from confounding by the maternal genome (2c). Paths 3c and 4c indicate how paternally transmitted alleles in the offspring and father may result in a biased causal estimate. Panel (d) illustrates an offspring genotype-by-proxy MR approach where spousal information (i.e. father’s genotype) is utilized to proxy offspring genotype (1d), which in turn proxies the offspring perinatal exposure (2d). Thus, paternal genetic variants can be used as instrumental variables to estimate the causal effect of an offspring’s perinatal exposure on a maternal outcome (as indicated by the path in bold). This obviates the need for offspring genotype (indicated by denoting offspring genotype as a latent unobserved variable in the figure). Under random mating, paternal and maternal genotypes should be uncorrelated (3d). The design also assumes the absence of pleiotropic paths from the offspring genome to the maternal phenotype (4d) and direct effects of the paternal genotype on maternal phenotype (5d).

Establishing whether observational associations are causal is challenging, especially in the perinatal context, owing to the complex intergenerational interplay of genetic and environmental factors that can confound the relationship. Additionally, observational associations can reflect reverse causality and bidirectional influences; e.g., poor maternal health during pregnancy can affect the offspring’s perinatal health, which in turn may further deteriorate maternal health. One method to aid in the triangulation of evidence is Mendelian randomization (MR). MR uses genetic variants as instrumental variables to test for a causal effect of an exposure on medically relevant outcomes [11]. MR is robust to some sources of confounding, reverse causality and many types of bias. However, it relies on a number of assumptions that have previously been discussed at length [12, 13] (Fig. 1a and Supplementary Material).

We use the potential causal effect of fetal growth (e.g. small/large for gestational age as measured by offspring birth weight) on maternal postnatal health as an illustrative example. Here, MR can be used for causal hypothesis testing by using offspring genetic variants to proxy offspring birth weight. However, in addition to the usual limitations inherent with MR studies, the correlation between maternal and offspring genotypes can lead to inconsistent causal effect estimates through violation of the independence assumption (Fig. 1a). Conditioning on maternal genotype (i.e. at the same loci as in the offspring; as indicated in Fig. 1b) is one way to block this path of maternal confounding [12]. We refer to this approach as “MR with adjustment for maternal genotype.” Alternatively, the availability of genomic data on parent–offspring trios and/or mother–offspring dyads permits the identification of paternally transmitted alleles, which should also avoid confounding by the maternal genome (Fig. 1c; referred to as “MR using paternally transmitted alleles”) [14]. As an example, Chen *et al.* constructed paternally transmitted genetic scores for birth weight in 10 734 genotyped mother–offspring pairs and found these were associated with increased maternal systolic blood pressure during pregnancy and reduced gestational duration [15]. Similarly, Petry *et al.* identified an association between paternally transmitted fetal *IGF2* alleles and increased maternal glucose concentrations in the third trimester of pregnancy using 1160 parent–offspring trios [4]. However, these approaches are difficult to perform at scale because of the paucity of genotyped parent–offspring dyads and trios across the world’s cohorts [16]. Consequently, this limits the utility and broader application of MR in investigating the causal effect of offspring perinatal traits on maternal health outcomes.

In this article, we introduce a new approach “offspring genotype-by-proxy” MR, which can be employed in the absence of offspring genetic information, to complement existing methods in the triangulation of causal inference. This framework exploits studies, such as UK Biobank (UKB) that have information on tens of thousands of genome-wide genotyped spousal pairs. We also describe how this can be implemented using genotype data from unrelated individuals and linked health record data from their offspring and spouses. As explained in more detail below, the method leverages the correlation between parental and offspring genotypes, using paternal genotypes to capture the direct effect of their offspring’s genotype on their offspring’s own exposure. We use causal graphs, asymptotic distribution theory, and simulation studies to compare offspring genotype-by-proxy MR with existing approaches based on genotyped mother–offspring dyads and trios in terms of statistical power and (in)consistency due to horizontal pleiotropy. In our explication, we focus on putative causal effects of offspring perinatal exposures (e.g. birth weight or small/large for gestational age) on their mother’s future postnatal health, noting that the assumptions of our approach are unlikely to hold for many postnatal and later life offspring phenotypes.

## Development and application

### Offspring genotype-by-proxy Mendelian randomization

The offspring genotype-by-proxy MR approach enables MR studies of the causal effect of offspring perinatal traits on their mother’s outcomes when offspring genotypes are not available, but paternal genotypes are. To understand why the offspring genotype-by-proxy approach would be informative, consider Fig. 1d, which illustrates a credible way in which causal estimates of perinatal offspring traits on maternal outcomes can be obtained in the absence of offspring genotypes. As fathers transmit half their alleles to their offspring, it follows that paternal genotype will proxy offspring genotype, which in turn will proxy offspring phenotype (path 1d and 2d in Fig. 1d). In the absence of horizontal pleiotropy between the paternal genotype and maternal outcome (and between offspring genotype and maternal outcome), this allows testing for a causal effect of the perinatal offspring trait on the maternal outcome. Using paternal genotype also obviates the requirement for individual-level genotyped mother–offspring pairs, although at the cost of a decrement in power compared to performing MR with adjustment for maternal genotype or MR using paternally transmitted alleles in genotyped mother–offspring pairs or parent–offspring trios (discussed in greater detail below). It is important to emphasize that paternal genetic variants are used as instrumental variables (as opposed to maternal genetic variants) to minimize confounding from maternal genotype. For example, using maternal genotype to proxy offspring genetic variants related to birthweight would also likely include variants that pleiotropically influence maternal blood pressure and glucose levels. The offspring genotype-by-proxy MR approach could be employed in combination with “traditional” MR analyses involving mother–offspring dyads (i.e. MR with adjustment for maternal genotype) or parent–offspring trios (i.e. MR using paternally transmitted alleles) and other informative designs to augment power and aid in the triangulation of causal evidence [17]. We contrast these three approaches in Table 1, including their application in one- and two-sample MR and potential sources of bias, as subsequently discussed in greater detail.

**Table 1:** Comparison of three different MR approaches for estimating the causal effect of a perinatal offspring exposure on the mother’s future postnatal health.

Method	Offspring genotype-by-proxy MR	MR with adjustment for maternal genotype	MR using paternally transmitted alleles
Description of the method (one-sample MR)	Offspring genotype is proxied using paternal genotype at the genetic variant for the exposure in the father. MR is then performed using paternal genotype, offspring exposure, and maternal health outcome, as would be possible with genotyped spousal pairs and offspring phenotype information.	MR is performed using offspring genotype as the instrument, offspring exposure, and maternal health outcome, with analyses adjusted for maternal genotype (at the same loci as in the offspring) as would be possible with genotyped mother–offspring pairs.	Paternal transmitted alleles are identified based on direct comparison of genotyped or local haplotype sharing using dense genome-wide trio data. MR is performed using the transmitted paternal alleles as the instrument, offspring exposure, and maternal health.
Data required (one-sample MR)	Genotyped fathers (or spousal pairs) with offspring exposure traits and maternal health outcomes.	Genotyped mother–offspring dyads with offspring exposure traits and maternal health outcomes.	Genotyped parent–offspring dyads or trios with offspring exposure traits and maternal health outcomes.
Description of the method (two-sample MR)	SNP–exposure association estimates concerning the effect of offspring genotype on offspring exposure can be obtained from general GWAS summary statistics (assuming that there are no maternal/paternal effects at the locus). SNP–outcome association needs to be obtained from the genotyped father under study, i.e. the association between paternally proxied offspring genetic variants and the maternal outcome.	SNP–exposure association estimates concerning the effect of offspring genotype on offspring exposure. SNP–outcome association estimates the effect of offspring genotype on maternal outcome (conditional on maternal genotype).	SNP–exposure association estimates concerning the effect of paternally transmitted alleles on offspring exposure. SNP–outcome association estimates the effect of paternally transmitted alleles on maternal outcome.
Data required (two-sample MR)	SNP–exposure sample requires its own genotype and phenotype. SNP–outcome sample requires genotyped parous spousal pairs or genotyped fathers with linked spousal phenotypic data.	SNP–exposure sample requires its own genotype and phenotype. SNP–outcome sample requires mother–offspring dyads.	Genotyped mother–offspring or father–offspring dyads or trios are required to identify paternally transmitted alleles for both the SNP–exposure and SNP–outcome associations.
Potential sources of bias	Assortative mating on the exposure (or correlated traits) may introduce confounding through maternal genotype. Horizontal pleiotropy of paternal genetic variants on the maternal outcome (i.e. through paternal phenotypes). Horizontal pleiotropy through offspring genetic variants.	Confounding through the paternal genome Horizontal pleiotropy of offspring genetic variants.	Horizontal pleiotropy of paternal genetic variants (at paternally transmitted alleles) for the exposure on the maternal outcome (i.e. through paternal phenotypes). Horizontal pleiotropy of offspring genetic variants.
Limitations	Decreased power due to proxying offspring genotype.	Requires genotyped maternal–offspring dyads.	Requires genotyped parent–offspring dyads or trios.

The three MR approaches include (1) Offspring genotype-by-proxy MR, (2) MR with adjustment for maternal genotype, and (3) MR using paternally transmitted alleles. We provide a description of each approach within the one- and two-sample MR framework including the minimum data required for each. Note that for causal hypothesis testing (as opposed to causal effect estimation) only the SNP–outcome association is required. The potential source of bias and limitation for each of the three MR approaches are listed.

Given the increasing availability of datasets such as the UK Biobank that (incidentally) include tens of thousands of genome-wide genotyped spousal pairs [18], the offspring genotype-by-proxy MR approach could augment causal analyses of perinatal offspring exposures on their mother’s outcomes. To see an empirical application of the method, cf [19]. In addition, we note that offspring genotype-by-proxy MR can also be implemented in parous fathers even when genetic data is not available for their spouse (i.e. their offspring’s biological mother). Instead, large genetic datasets where data linkage to population-based registries and health records is available would permit implementation of the offspring genotype-by-proxy MR approach at a scale currently unattainable for MR approaches based on parent–offspring trios and/or mother–offspring dyads. For example, the Medical Birth Registry of Norway, which comprises data on the mother, father, and newborn for all births in the country after 16 weeks’ gestation, can be linked to genotyped participants in HUNT [20] as well as national epidemiological registries that allow investigation of later life health outcomes [21]. Data linkage of nation-wide, epidemiological registries for individuals as well as their parents, spouses, children, and siblings is also available in other countries, e.g. FinRegistry [22]. The ability to link such registries to large genetic datasets containing (at least a subset of) the same individuals [e.g. The FinnGen study (https://www.finngen.fi/en), which contains genomic data with linkage to health register data of 500 000 Finnish biobank participants] allow for similar analyses to be conducted. Subsequently, a large-scale investigation of causal effects of perinatal offspring traits on their parents’ health, such as that proposed here, could be conducted while only requiring genotype information for a single individual in each parent–offspring trio. In contrast, both MR conditional on maternal genotype and MR using paternally transmitted alleles require genotyped mother–offspring or father–offspring dyads, even when implemented in the two-sample design (Table 1). As the offspring genotype-by-proxy MR approach relies on the assumption that any association between the paternally proxied offspring genetic variants for the exposure and maternal outcome operates through the offspring exposure, such analyses need to be performed in individuals who have not only had an offspring, but where the offspring exposure is present.

### Assumptions of offspring genotype-by-proxy MR

**Figure 2:**
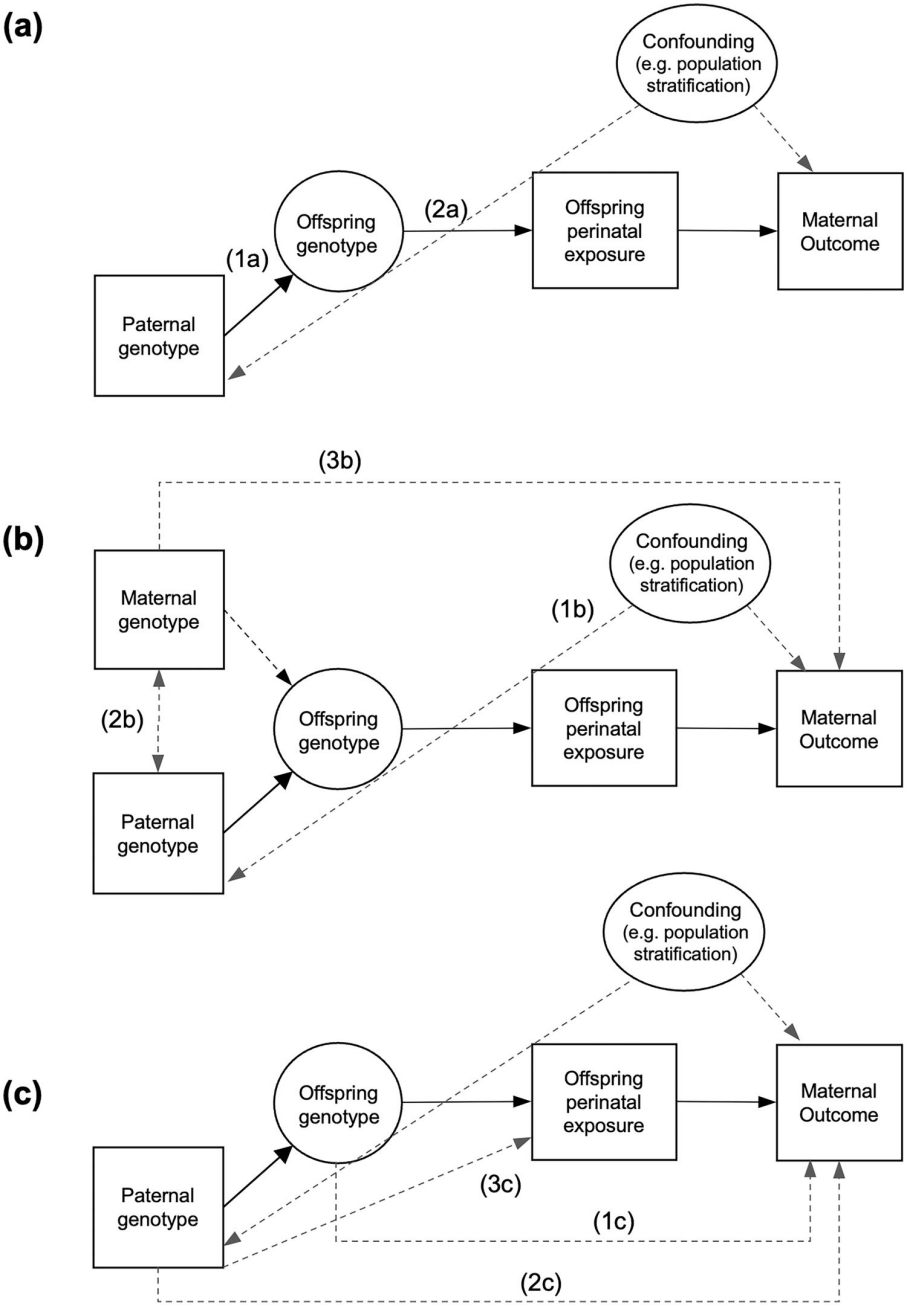
Causal diagram illustrating the offspring genotype-by-proxy MR approach and its underlying assumptions. Panel (a) illustrates the relevance assumption which requires correct matching of spousal pairs and for paternal genotype to be from the biological father of the offspring (path 1a) and the proxied offspring genetic variants to be statistically robustly associated with the exposure in the relevant population (path 2a). Panel (b) highlights the independence assumption whereby there are no confounders of the paternal genotype–maternal outcome relationship (1b). Further, genetic variants for the perinatal exposure in the father should not be associated with genetic variants in the mother (e.g. as might be the case under assortative mating on the exposure; path 2b) as this may create a path to maternal outcome if correlated variants in the mother have effects on her outcome (path 3b). Part (c) illustrates the exclusion restriction assumption which requires that no causal path exists from the paternal genotype (which is used to proxy the offspring genotype) to the maternal outcome (paths 1c and 2c) other than through the offspring exposure. While dynastic or parent of origin effects from paternal genotype to offspring exposure (path 3c) could be used to proxy the offspring exposure, these may be more likely be correlated with or exert effects on maternal phenotypes and may result in violation of the MR assumptions.

Causal hypothesis testing using the offspring genotype-by-proxy MR approach involves the same three core assumptions as conventional MR analyses; however, there are additional nuances in this context (Fig. 2). A detailed discussion of these assumptions is provided in the Supplementary Material. The relevance assumption requires that the genetic instruments are robustly associated with the exposure of interest in the relevant population. In the offspring genotype-by-proxy approach, this depends upon the accuracy of spousal matching and on the statistical strength of association between offspring genotype and offspring exposure (Fig. 2a). The independence assumption in MR requires no confounding between the genetic variants and outcomes of interest (Fig. 2b). This may be violated by confounding through the maternal genome, as maternal genotype will be correlated with offspring genotype (through transmission) and may also plausibly influence the maternal outcome. In the offspring genotype-by-proxy MR approach, we utilize the paternal genotype to proxy the offspring genotype, which should be uncorrelated with maternal genotype in the absence of assortative mating and therefore protect MR analyses from confounding by the maternal genome. This relies on the assumption of random mating for the exposure (and correlated traits). While spouses are extremely unlikely to assort primarily on perinatal traits, it is possible that perinatal exposures could be indirectly influenced by parental phenotypes where assortment is more likely. This can be mitigated by selecting genetic instruments that are unlikely to exhibit indirect genetic effects. Lastly, the exclusion restriction assumption stipulates that the genetic instrument must only be potentially associated with the outcome through the exposure. It is therefore assumed that there is no directed path between paternal genetic variants and maternal outcome other than that going through the offspring exposure (Fig. 2c). This includes the assumption of no pleiotropic paths from offspring genotype to maternal outcome (Fig. 2c, path 1c) and from paternal genotype to the maternal outcome through a paternal phenotype (Fig. 2, path 2c). We note that many of these assumptions are likely to be violated if this approach were applied to postnatal/later life offspring exposures including the absence of assortative mating on the exposure in the parental generation, and the absence of potential effects of the exposure phenotype in the father on the maternal outcome.

### Exploring violation of the MR assumptions

#### Inconsistency and bias of instrumental variable estimators in the presence of horizontal pleiotropy and weak instruments

We explore the consequences of violation of the exclusion restriction assumption by way of horizontal pleiotropy through either the offspring or the paternal genome. Following asymptotic theory, we derived the large sample properties of the IV estimator for each of the three different MR approaches (derivations provided in the Supplementary Material). We assume the absence of assortative mating and confounding from maternal genotype (noting that in the absence of assortative mating, all three approaches are robust to confounding from the maternal genotype). All three MR approaches yield inconsistent IV estimates when the assumption of no effect of offspring and/or paternal genetic effects on the maternal outcome is violated (i.e. horizontal pleiotropy). In the presence of pleiotropy through the offspring genotype, the probability limit of the IV estimator differed from the population-level causal effect. For all three approaches, this is equal to the effect of offspring genotype on maternal outcome divided by the effect of offspring genotype on offspring exposure. However, in the presence of pleiotropy through the paternal genotype, the probability limit of the IV estimator differed between the three approaches. The magnitude of difference between the probability limit of the IV estimator and the population-level causal effect was largest for the offspring genotype-by-proxy MR design, equal to twice the effect of paternal genotype on maternal outcome divided by the effect of offspring genotype on offspring exposure. This was reduced by a factor of ½ for MR using paternally transmitted alleles and by a factor of ¼ for MR with adjustment for maternal genotype. Thus, comparison of causal effect estimates from more than one approach would allow for estimation of the degree of pleiotropy through the paternal genome.

We complement the asymptotic derivations with data simulations to demonstrate the effect of realistic scenarios of horizontal pleiotropy and weak instruments on causal effect estimates (methods for data simulations provided in the Supplementary Material). When simulating pleiotropy through the offspring genotype, all three approaches resulted in a biased causal estimate (exaggerating the true positive causal effect and producing a non-null positive effect when the true effect was null) of similar magnitude (Table 2). In comparison, pleiotropy through the paternal genome resulted in biased causal estimates in all approaches, with the magnitude of bias largest in the offspring by proxy MR approach, followed by the paternal transmitted haplotype approach. For both sources of pleiotropy, the magnitude of bias increased as the strength of pleiotropy increased (from either the offspring or the paternal genotype). The magnitude of bias also increased as instrument strength decreased for all three approaches (as demonstrated by decreased strength of association between offspring genotype and offspring exposure). We provide simulation results demonstrating that all three approaches are robust to confounding from the maternal genotype in Supplementary Table 1, in the supplementary material.

**Table 2:** The impact of horizontal pleiotropy and weak instruments on the causal effect estimate of a perinatal offspring exposure on a maternal outcome using the three Mendelian randomization (MR) approaches.^a^

Simulated causal effect size	% variance in the offspring exposure explain by the offspring genetic variant	% variation in maternal outcome explained by pleiotropy from		Difference in means of maternal outcome (in standard deviations units) per 1 SD increase in offspring exposure that the IV is testing (SE: average model SE)		
		**Offspring genetic variant**	**Paternal genetic variant**	**(1) Offspring genotype-by-proxy MR**	**(2) MR with adjustment for maternal genotype**	**(3) MR using paternally transmitted alleles**
0.2	2%	0	0	0.20 (0.06)	0.20 (0.04)	0.20 (0.04)
0.2	2%	1%	0	0.91 (0.08)	0.91 (0.04)	0.90 (0.05)
0.2	2%	5%	0	1.79 (0.12)	1.78 (0.07)	1.78 (0.08)
0.2	2%	0	1%	1.62 (0.11)	0.67 (0.04)	0.91 (0.05)
0.2	2%	0	5%	3.37 (0.21)	1.25 (0.05)	1.78 (0.08)
0.2	0.5%	0	0	0.19 (0.13)	0.20 (0.07)	0.20 (0.09)
0.2	0.5%	1%	0	1.65 (0.23)	1.62 (0.13)	1.63 (0.16)
0.2	0.5%	5%	0	3.40 (0.45)	3.38 (0.25)	3.39 (0.31)
0.2	0.5%	0	1%	3.08 (0.41)	1.15 (0.10)	1.63 (0.16)
0.2	0.5%	0	5%	6.62 (0.88)	2.33 (0.17)	3.40 (0.31)
0	2%	0	0	0.00 (0.06)	0.00 (0.04)	0.00 (0.04)
0	2%	1%	0	0.71 (0.08)	0.71 (0.04)	0.71 (0.05)
0	2%	5%	0	1.59 (0.12)	1.58 (0.07)	1.59 (0.08)
0	2%	0	1%	1.42 (0.11)	0.47 (0.04)	0.71 (0.05)
0	2%	0	5%	3.19 (0.21)	1.06 (0.05)	1.59 (0.08)
0	0.5%	0	0	0.00 (0.13)	0.00 (0.07)	0.00 (0.09)
0	0.5%	1%	0	1.43 (0.23)	1.42 (0.13)	1.43 (0.16)
0	0.5%	5%	0	3.23 (0.45)	3.18 (0.25)	3.19 (0.31)
0	0.5%	0	1%	2.87 (0.41)	0.95 (0.10)	1.42 (0.16)
0	0.5%	0	5%	6.40 (0.87)	2.11 (0.17)	3.17 (0.30)

a The approaches were (1) MR using offspring genotype by proxy, (2) MR involving offspring genotype conditional on maternal genotype, and (3) MR utilizing the transmitted paternal haplotype. Data simulations were based on 50 000 parent–offspring trios across 1000 replicates. The causal effect is the difference in mean maternal outcome in standard deviation units per unit increase in offspring exposure. We show results for a true non-null (β_XY_ = 0.2) causal effect and for a null effect (β_XY_ = 0), where the effect of confounders on the exposure and the outcome is (β_UX_ = β_UY_ = 0.1). Instrument strength is proportional to variance in the offspring exposure explained by the offspring genetic variant, which varies from 2% to 0.5% to represent strong and weak instruments. We varied the proportion of phenotypic variance in the maternal outcome explained by pleiotropy from the offspring and paternal genotypes to range from 0% to 5%. SD, standard deviation; SE, standard error.

#### Misclassification of spousal pairs

Matching of spousal pairs based on demographic information, such as in the UK Biobank, is subject to misclassification. Further, correct matching of spousal pairs does not necessitate that both individuals are the biological parents of any reported offspring. We considered the consequences of (accidental) inclusion of incorrectly matched spousal pairs (or correctly matched spousal pairs that are not biological parents) on power for causal hypothesis testing and bias in the causal estimate. We performed a simulation study, varying the proportion of randomly misclassified spouses (0%–40% of spousal pairs) and investigated two misclassification scenarios (Supplementary Fig. 1, in the supplementary material): (1) a male who is genetically unrelated to the offspring under consideration is incorrectly used to proxy the offspring exposure; (2) a genetically unrelated female is incorrectly paired with the father and his offspring. Full details for the data generation and simulation study can be found in the Supplementary Material, and full simulation results in Supplementary Table 2, in the supplementary material.

The simulation shows that the effect of spousal pair misclassification depends on the true relationship between offspring and putative parents (assuming the exposure is reported by the true parent; Table 3). In the first scenario, misclassification was associated with decreased strength of association between the instrument and the exposure; however, causal estimates were largely unbiased if instruments were strong. In scenario 2, the causal effect attenuated toward the null as misclassification increased. In this scenario instrument strength was not impacted as misclassification increased. In both scenarios, statistical power decreased as instrument strength decreased, and spousal pair misclassification increased.

**Table 3:** The impact of spousal pair misclassification on the instrumental variables estimate of offspring exposure on maternal outcome using offspring genotype-by-proxy MR.^a^

% variance in the offspring exposure explained by offspring genotype	Proportion of misclassified spousal pairs	Difference in means of maternal outcome per 1SD increase in offspring exposure	Average model standard error	Power	*F*-statistic	Coverage
**Misclassification scenario 1: male not related to offspring**						
0.5%	0	0.194	0.12	0.41	62	0.96
0.5%	0.1	0.190	0.14	0.34	50	0.95
0.5%	0.2	0.193	0.15	0.31	40	0.96
0.5%	0.3	0.186	0.18	0.22	30	0.96
0.5%	0.4	0.183	0.21	0.20	23	0.97
2%	0	0.198	0.06	0.91	251	0.96
2%	0.1	0.197	0.07	0.82	202	0.94
2%	0.2	0.198	0.07	0.75	160	0.96
2%	0.3	0.195	0.09	0.65	122	0.95
2%	0.4	0.196	0.10	0.51	91	0.96
**Misclassification scenario 2: female not related to offspring**						
0.5%	0	0.194	0.12	0.41	62	0.96
0.5%	0.1	0.172	0.12	0.33	62	0.95
0.5%	0.2	0.156	0.12	0.29	62	0.97
0.5%	0.3	0.133	0.12	0.21	62	0.94
0.5%	0.4	0.113	0.12	0.17	63	0.93
2%	0	0.198	0.06	0.91	251	0.96
2%	0.1	0.177	0.06	0.81	250	0.93
2%	0.2	0.159	0.06	0.75	250	0.91
2%	0.3	0.137	0.06	0.63	250	0.84
2%	0.4	0.118	0.06	0.49	252	0.75

a Data simulations were based on 50 000 parent–offspring trios across 1000 replicates. A causal effect of β_XY_ = 0.2 was used, i.e. the difference in mean maternal outcome in standard deviation units per unit increase in offspring exposure. The strength of confounding between the offspring exposure and the maternal outcome was set at β_UX_ = β_UY_ = 0.5. The variance explained by the offspring genetic variant in the offspring exposure varies from 2% to 0.5%. In scenario (1), a male who is genetically unrelated to the offspring under consideration is incorrectly used as a proxy for the offspring exposure, while in scenario (2), a genetically unrelated female is incorrectly paired with the father and his offspring. SD, standard deviation.

### Application of offspring genotype-by-proxy MR within the gene-by-environment MR framework

Application of the MR gene-by-environment (GxE) by stratifying on spousal parity (i.e. inclusion and comparison to spousal pairs who do not have children) can provide evidence for violation of the independence and exclusion restriction assumptions and enable estimation of the causal effect of the exposure on the outcome, correcting for such violations [23–25]. Here, nulliparous spousal pairs effectively act as negative controls, allowing for the identification of heterogeneity in effect estimates owing to non-causal influences. For example, if the offspring exposure is causal for the maternal health outcome, then genetic instruments for the exposure in the father should only be associated with maternal outcomes in spousal pairs who have shared an offspring. Conversely, the “offspring” exposure could not plausibly influence the outcome in nulliparous pairs. The presence of a (spurious) “causal” association between the genetic instrument and the outcome in nulliparous spousal pairs can be used to quantify the degree of horizontal pleiotropy and confounding present in the analysis, and to subsequently correct any causal estimates for pleiotropy in parous spousal pairs. Interestingly, utilizing GxE interactions in nulliparous spousal pairs is only possible when proxying offspring genotype using paternal genotypes as opposed to the other MR approaches discussed here, which by definition involve parous mothers. Finally, we note that there may be opportunities afforded by broken partnerships in modeling causal effects where these data are available (and conversely interpretational complexities where they are not), with similar designs proposed using adopted versus biological relatives [26].

### Power of the offspring genotype-by-proxy MR approach

**Figure 3:**
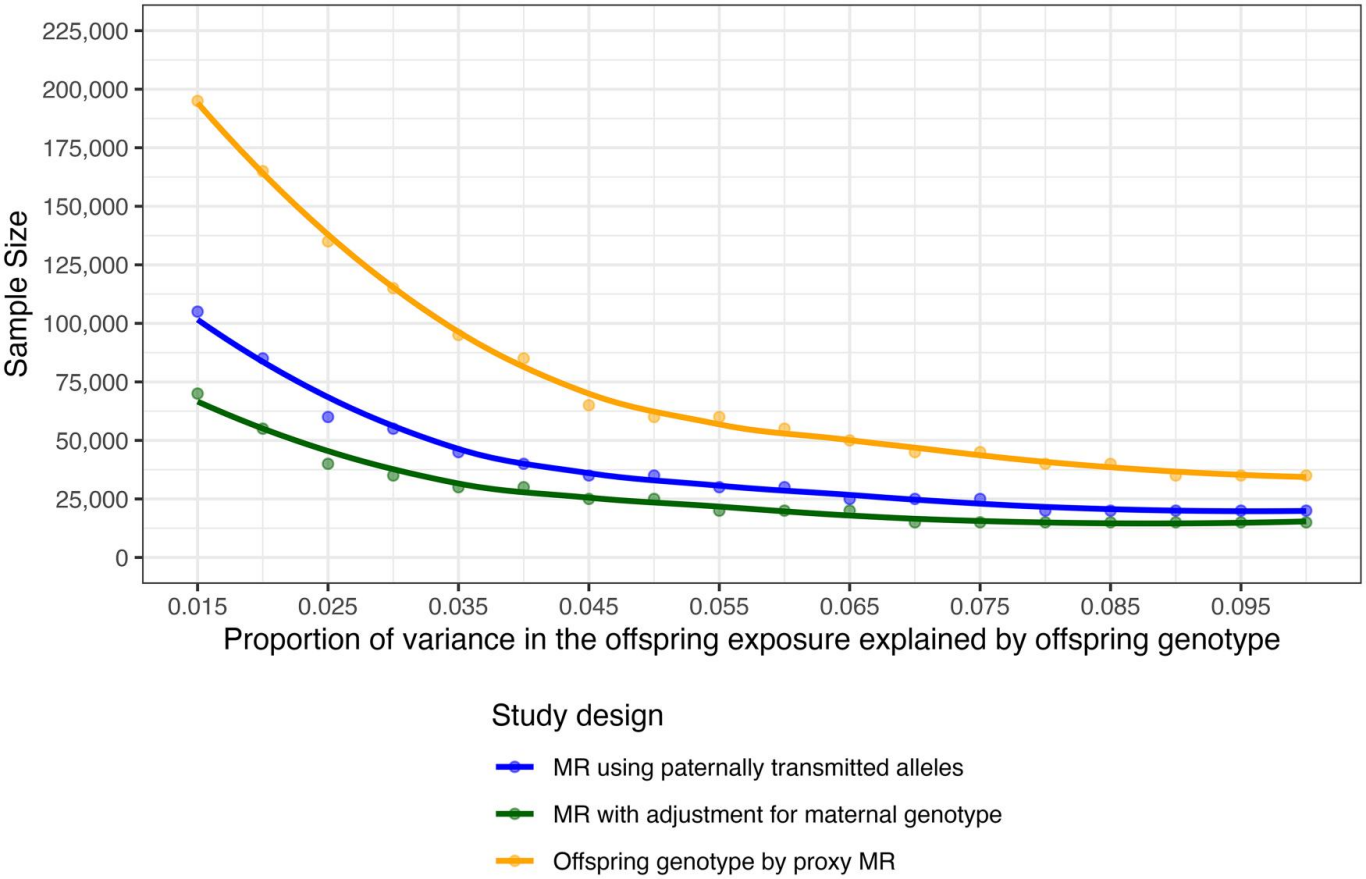
Illustrative examples of the minimum sample size required for 80% power to detect a causal effect estimate of offspring exposure on maternal outcome using three Mendelian randomization (MR) approaches estimated using simulation. The approaches were (1) Offspring genotype-by-proxy MR, (2) MR with adjustment for maternal genotype, and (3) MR using paternally transmitted alleles. The sample size is presented for varying proportions of variance in the offspring exposure explained by the offspring genetic instrument. For each approach, a single observational unit represents a genotyped parent–offspring trio (even if not all are needed for the analysis). Power calculations were performed assuming a type 1 error rate of α = 0.05 and a causal effect size of β_XY_ = 0.1. The causal effect is the difference in mean maternal outcome in standard deviation units per standard deviation increase in offspring exposure. The strength of confounding between the offspring exposure and maternal outcome was set at β_UX_ = β_UY_ = 0.1, and estimates were based on the mean of 1000 simulation replicates.

Genetic variants typically only explain small amounts of variation in exposure variables and thus require very large sample sizes to detect causal effects with appreciable power [27]. This limitation is exacerbated in the offspring genotype-by-proxy MR approach, with the proportion of variation in the offspring exposure explained by the paternal genotype reduced by a factor of 0.25 relative to that explained by the offspring’s own genotype. We used simulations to contrast power to estimate a causal effect for the three MR approaches (methods for the data simulations outlined in the Supplementary Material) and confirm these using asymptotic power calculations [27] (Supplementary Table 3, in the supplementary material). Figure 3 displays the minimum sample size required to obtain 80% power (type I error rate α = 0.05) to detect a causal effect using each of the three study designs, assuming a true non-null causal effect of β_XY_ = 0.1. For the offspring genotype-by-proxy MR approach, the variance explained by the genetic instruments is reduced by a factor of 1/4 relative to that of the mother–offspring pairs approach. Subsequently, our approach will be less powerful than those based on similar-sized samples of genotyped mother–offspring pairs. The corollary is that, to have enough power to resolve causal effects on its own given current sample sizes, offspring genotype-by-proxy MR would need to be employed in very large samples of spousal pairs using allelic scores that explain considerable proportions of variance in the offspring exposure variable. Despite this, as there is a paucity of large cohorts of genotyped mother–offspring pairs and parent–offspring trios around the world, the method could provide a complementary approach to causal inference owing to the availability of large numbers of genome-wide genotyped spousal pairs in publicly available genetic datasets such as the UK Biobank as well as large population-based biobanks with linked health record data for first-degree relatives.

### Estimation of causal effects: one- versus two-sample MR

**Figure 4:**
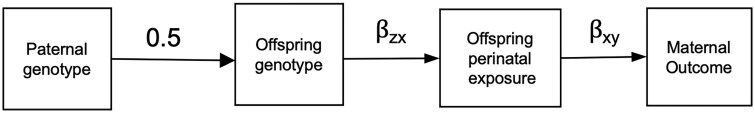
Estimating the causal effect of an offspring perinatal exposure on a maternal health outcome using offspring genotype-by-proxy Mendelian randomization (MR). $\beta_{\mathrm{zx}}$ represents the effect of offspring genotype on the offspring exposure, and $\beta_{\mathrm{xy}}$ is the causal effect of offspring exposure on the maternal outcome. In the absence of an offspring genotype, the paternal genotype is used as a proxy. In one-sample MR, the genetic instrument, exposure, and outcome are measured in the same sample, and the causal effect can be estimated using two-stage least squares. In the first stage, the offspring exposure is predicted using the proxied offspring genotype i.e. using paternal genotype. The causal effect estimate is then obtained by regressing the maternal outcome on the predicted offspring exposure and calculating appropriate standard errors. Alternatively, a Wald ratio using summary level data from different samples of the same underlying population can be calculated using the SNP–exposure association (e.g. between paternal genetic variants and the offspring exposure) and estimates of the SNP–outcome association (between paternal genetic variants and their spouse’s outcome).

In the offspring genotype-by-proxy MR design, it is essential to match the genetic variants for the perinatal offspring exposure in the father (i.e. genetic variants in the father for their own perinatal trait) with the maternal outcome. The most intuitive way of estimating the causal effect would be in a single sample using two-stage least squares. The causal effect can also be estimated in two-sample MR using the Wald ratio, and we present a diagram illustrating estimation of the causal effect in Fig. 4. While evaluating the effect of the proxied, offspring genotype on the maternal outcome is sufficient for causal hypothesis testing, point estimate identifying assumptions are required for causal effect estimation. Specifically, in the two-sample context, we assume that any association between own genotype and own exposure is consistent across generations, with the degree to which this is plausible dependent on the causal hypothesis being tested.

As the single nucleotide polymorphism (SNP)–exposure association aims to capture the effect of offspring genotype on offspring exposure, it is also possible to use the association between own genotype and own exposure from general genome-wide association study (GWAS) summary statistics (assuming that there are no maternal/paternal effects at the locus and no generational differences in the association between own genotype and own exposure). Conversely, the SNP–outcome association needs to be obtained from the spousal pairs under study, i.e. the association between paternal genetic variants and the maternal outcome as described in Fig. 4. The resulting SNP–outcome association is equal to half that would be estimated using offspring genotype, i.e. ${{0.5\;\times \hat{\beta }}_{\mathrm{zx}}\;\times \hat{\beta }}_{\mathrm{xy}}$. Consequently, a correction (multiplication by two) needs to be applied to obtain an estimate of the causal effect of the offspring exposure, i.e. ${\hat{\beta }}_{\mathrm{xy}}=\frac{{(0.5\;{\hat{\beta }}_{\mathrm{zx}}\hat{\beta }}_{\mathrm{xy}})\times 2}{{\hat{\beta }}_{\mathrm{zx}}}$. The corresponding standard error can be estimated using the first-order terms of the delta method, i.e. ${\hat{SE}}_{{\hat{\beta }}_{\mathrm{xy}\;}}=\frac{2SE({0.5\;{\hat{\beta }}_{\mathrm{zx}}\hat{\beta }}_{\mathrm{xy}})}{{\hat{\beta }}_{\mathrm{zx}}}$. This can be extended to the case where causal effects are estimated using more than one SNP (i.e. using an inverse variance weighted estimator). The use of two-sample MR may improve the accuracy of causal estimates by leveraging larger sample sizes for the SNP–exposure association and mitigating any effect of weak instrument bias [28]. In addition, the robustness of causal estimates can be investigated using MR sensitivity approaches such as weighted median, weighted mode, and MR egger, the latter of which is not appropriate with overlapping samples [29].

## Conclusion

We have developed an MR framework that can be used to estimate the causal effect of a perinatal offspring trait on their mother’s health outcomes in the absence of offspring genotype data. We present a comparison with other MR approaches based on genotyped mother–offspring pairs and demonstrate that all methods are biased in the presence of pleiotropy through the offspring or paternal genome, the latter of which may be identified by comparing effect estimates across approaches. Unlike most traditional MR studies, our framework includes an inbuilt control for violation of core assumptions through the inclusion and comparison of causal effect estimates derived from spousal pairs who do not have children.

As there is a dearth of large cohorts of genotyped mother–offspring pairs/parent–offspring trios, our method offers an opportunity to provide complementary information on the existence of offspring causal effects owing to the growing availability of large numbers of genome-wide genotyped spousal pairs in publicly available genetic datasets such as the UK Biobank.

## Ethics approval

This project received ethical approval from the Institutional Human Research Ethics committee, University of Queensland (Approval Number 2019002705).

## Supplementary Material

dyag030_Supplementary_Data

## Data Availability

No new data were generated or analyzed in support of this research.
