# Supplementary material for: Utilizing offspring genotype-by-proxy Mendelian randomization to investigate the causal effect of offspring perinatal traits on maternal health
Source: Int J Epidemiol. 2026 Mar 9;55(2):dyag030. doi: 10.1093/ije/dyag030 (PMC13017718; doi:10.1093/ije/dyag030)
Supplement: dyag030_Supplementary_Data [file dyag030_supplementary_data.zip › Supplementary_Data.pdf]

Utilising offspring genotype by proxy Mendelian randomization to investigate the causal effect of offspring perinatal traits on maternal health

Alesha A Hatton[1], Caroline Brito Nunes[1], Deborah Lawlor[2,3], David M Evans[1,2,4*]

Affiliations
[1]Institute for Molecular Bioscience, The University of Queensland, St Lucia, QLD 4067, Australia
[2]MRC Integrative Epidemiology Unit, University of Bristol, Bristol, BS1 5DS, United Kingdom
[3]Population Health Science, Bristol Medical School, University of Bristol, Bristol, BS1 5DS, United Kingdom
[4]Frazer Institute, The University of Queensland, Woolloongabba, QLD 4102, Australia

*Corresponding author: David M Evans
d.evans1@uq.edu.au
Institute for Molecular Bioscience, The University of Queensland, 306 Carmody Rd, St Luci, QLD 4067, Australia

Supplementary Data

Table of Contents

SUPPLEMENTARY DATA **1**
Supplementary Tables 2
Supplementary Figures 6
Supplementary Methods 7
Assumptions of Mendelian Randomization studies of the causal effect of offspring perinatal traits on maternal health outcomes *7*
Assumptions of offspring genotype by proxy MR *8*
Inconsistency of instrumental variable estimators under pleiotropy *11*
Methods for data simulations *12*
References 15

Supplementary Tables

Table S1: Results from the simulation study on the impact of confounding through the maternal genotype on the instrumental variables estimate of offspring exposure on maternal outcome using the three Mendelian randomization (MR) approaches.

**Simulated causal effect size**	**Variance in the offspring exposure explain by the offspring genetic variant**	**Variance in maternal outcome explained by confounding from the maternal genetic variant**	**Difference in means of maternal outcome (in standard deviations units) per 1SD increase in offspring exposure that the IV is testing (SE: average model SE)**		
			**Offspring genotype by proxy MR**	**MR with adjustment for maternal genotype**	**MR using paternally transmitted alleles**
0.2	2%	0	0.2 (0.06)	0.2 (0.04)	0.2 (0.04)
0.2	2%	1%	0.2 (0.06)	0.2 (0.04)	0.2 (0.04)
0.2	2%	5%	0.2 (0.06)	0.2 (0.03)	0.2 (0.04)
0.2	0.50%	0	0.19 (0.13)	0.2 (0.07)	0.2 (0.09)
0.2	0.50%	1%	0.2 (0.13)	0.2 (0.07)	0.2 (0.09)
0.2	0.50%	5%	0.21 (0.13)	0.2 (0.07)	0.21 (0.09)
0	2%	0	0 (0.06)	0 (0.04)	0 (0.04)
0	2%	1%	0 (0.06)	0 (0.04)	0 (0.04)
0	2%	5%	-0.01 (0.06)	0 (0.04)	0 (0.04)
0	0.50%	0	0 (0.13)	0 (0.07)	0 (0.09)
0	0.50%	1%	0 (0.13)	0 (0.07)	0 (0.09)
0	0.50%	5%	-0.01 (0.13)	0 (0.07)	0 (0.09)

Table S2: Results from the simulation study investigating the magnitude of bias in the causal effect estimates and power to detect a causal effect under spousal pair misclassification.

**Misclassification scenario**	**True causal effect**	**Confounding**[a]	**Variance explained by offspring genotype**[b]	**Proportion of misclassified spousal pairs**	**Mean estimated causal effect**	**SE**[c]	**Power**[d]	**F-stat**[e]	**Coverage**[f]
1	0.2	0.50	0.5%	0	0.194	0.12	0.41	62	0.96
1	0.2	0.50	0.5%	0.1	0.190	0.13	0.34	50	0.95
1	0.2	0.50	0.5%	0.2	0.193	0.15	0.31	40	0.96
1	0.2	0.50	0.5%	0.3	0.186	0.17	0.22	30	0.96
1	0.2	0.50	0.5%	0.4	0.183	0.20	0.20	23	0.97
1	0.2	0.50	2.0%	0	0.198	0.06	0.91	251	0.96
1	0.2	0.50	2.0%	0.1	0.197	0.07	0.82	202	0.94
1	0.2	0.50	2.0%	0.2	0.198	0.07	0.75	160	0.96
1	0.2	0.50	2.0%	0.3	0.195	0.08	0.65	122	0.95
1	0.2	0.50	2.0%	0.4	0.196	0.10	0.51	91	0.96
2	0.2	0.50	0.5%	0	0.194	0.12	0.41	62	0.96
2	0.2	0.50	0.5%	0.1	0.172	0.12	0.33	62	0.95
2	0.2	0.50	0.5%	0.2	0.156	0.12	0.29	62	0.97
2	0.2	0.50	0.5%	0.3	0.133	0.12	0.21	62	0.94
2	0.2	0.50	0.5%	0.4	0.113	0.12	0.17	63	0.93
2	0.2	0.50	2.0%	0	0.198	0.06	0.91	251	0.96
2	0.2	0.50	2.0%	0.1	0.177	0.06	0.81	250	0.93
2	0.2	0.50	2.0%	0.2	0.159	0.06	0.75	250	0.91
2	0.2	0.50	2.0%	0.3	0.137	0.06	0.63	250	0.84
2	0.2	0.50	2.0%	0.4	0.118	0.06	0.49	252	0.75
1	0	0.50	0.5%	0	-0.006	0.13	0.04	62	0.96
1	0	0.50	0.5%	0.1	-0.010	0.14	0.05	50	0.95
1	0	0.50	0.5%	0.2	-0.006	0.16	0.03	40	0.97
1	0	0.50	0.5%	0.3	-0.014	0.18	0.04	30	0.96
1	0	0.50	0.5%	0.4	-0.016	0.21	0.03	23	0.97
1	0	0.50	2.0%	0	-0.002	0.06	0.04	251	0.96

1	0	0.50	2.0%	0.1	-0.003	0.07	0.06	202	0.94
1	0	0.50	2.0%	0.2	-0.001	0.08	0.04	160	0.96
1	0	0.50	2.0%	0.3	-0.005	0.09	0.05	122	0.95
1	0	0.50	2.0%	0.4	-0.004	0.11	0.04	91	0.96
2	0	0.50	0.5%	0	-0.006	0.13	0.04	62	0.96
2	0	0.50	0.5%	0.1	-0.008	0.13	0.05	62	0.95
2	0	0.50	0.5%	0.2	-0.004	0.13	0.04	62	0.96
2	0	0.50	0.5%	0.3	-0.008	0.13	0.05	62	0.95
2	0	0.50	0.5%	0.4	-0.006	0.13	0.04	63	0.96
2	0	0.50	2.0%	0	-0.002	0.06	0.04	251	0.96
2	0	0.50	2.0%	0.1	-0.003	0.06	0.06	250	0.94
2	0	0.50	2.0%	0.2	-0.001	0.06	0.04	250	0.96
2	0	0.50	2.0%	0.3	-0.003	0.06	0.05	250	0.95
2	0	0.50	2.0%	0.4	-0.002	0.06	0.05	252	0.95

[a] Confounding represents the strength of confounding between the offspring exposure and maternal outcome.

[b] Variance explained by offspring genotype is the proportion of variance in the offspring exposure explained by offspring genotype.

[c] The SE is the average model standard error of the estimated causal effect.

[d] Power is defined as power to detect a causal effect with a type 1 error rate 5%.

[e] F-stat is the F-statistic of the association between genetic instrument and offspring exposure.

[f] Coverage is defined as the proportion of simulations where the true causal effect lies within the 95% confidence interval for the estimand.

Table S3: Minimum sample size required for 80% power to detect a causal effect of an offspring exposure on maternal health outcome using offspring genotype by proxy Mendelian randomization (MR).

Sample size is presented for power calculations from asymptotic theory and data simulation. Power is defined as power to detect a causal effect with a type 1 error rate 5%.

			Results from asymptotic theory	Results from data simulations			
**True causal effect**	**Confounding** [a]	**Variance explained by offspring genotype** [b]	**Sample size** [c]	**Sample size** [c]	**Mean estimated causal effect**	**SE** [d]	**Power**
0.1	0.1	1.0%	313947	295000	0.10	0.04	0.81
0.1	0.1	1.5%	209296	205000	0.10	0.03	0.81
0.1	0.1	2.0%	156970	155000	0.10	0.03	0.82
0.1	0.1	2.5%	125574	135000	0.10	0.03	0.85
0.1	0.1	3.0%	104644	105000	0.10	0.03	0.81
0.1	0.1	3.5%	89694	95000	0.10	0.03	0.82
0.1	0.1	4.0%	78481	85000	0.10	0.03	0.82
0.1	0.1	4.5%	69760	70000	0.10	0.03	0.82
0.1	0.1	5.0%	62783	65000	0.10	0.03	0.84
0.1	0.1	5.5%	57075	60000	0.10	0.03	0.82
0.1	0.1	6.0%	52318	55000	0.10	0.03	0.81
0.1	0.1	6.5%	48293	50000	0.10	0.03	0.82
0.1	0.1	7.0%	44843	45000	0.10	0.03	0.83
0.1	0.1	7.5%	41853	40000	0.10	0.04	0.81
0.1	0.1	8.0%	39237	40000	0.10	0.03	0.83
0.1	0.1	8.5%	36928	40000	0.10	0.03	0.86
0.1	0.1	9.0%	34876	40000	0.10	0.03	0.88
0.1	0.1	9.5%	33040	35000	0.10	0.03	0.81
0.1	0.1	10.0%	31388	30000	0.10	0.04	0.81

[a] Confounding represents the strength of confounding between the offspring exposure and maternal outcome.

[b] Variance explained by offspring genotype is the proportion of variance in the offspring exposure explained by offspring genotype.

[c] Sample size is the minimum sample size required for at least 80% power to detect a causal effect (type 1 error rate 5%).

[d] SE is the average model standard error of the estimated causal effect.

Supplementary Figures

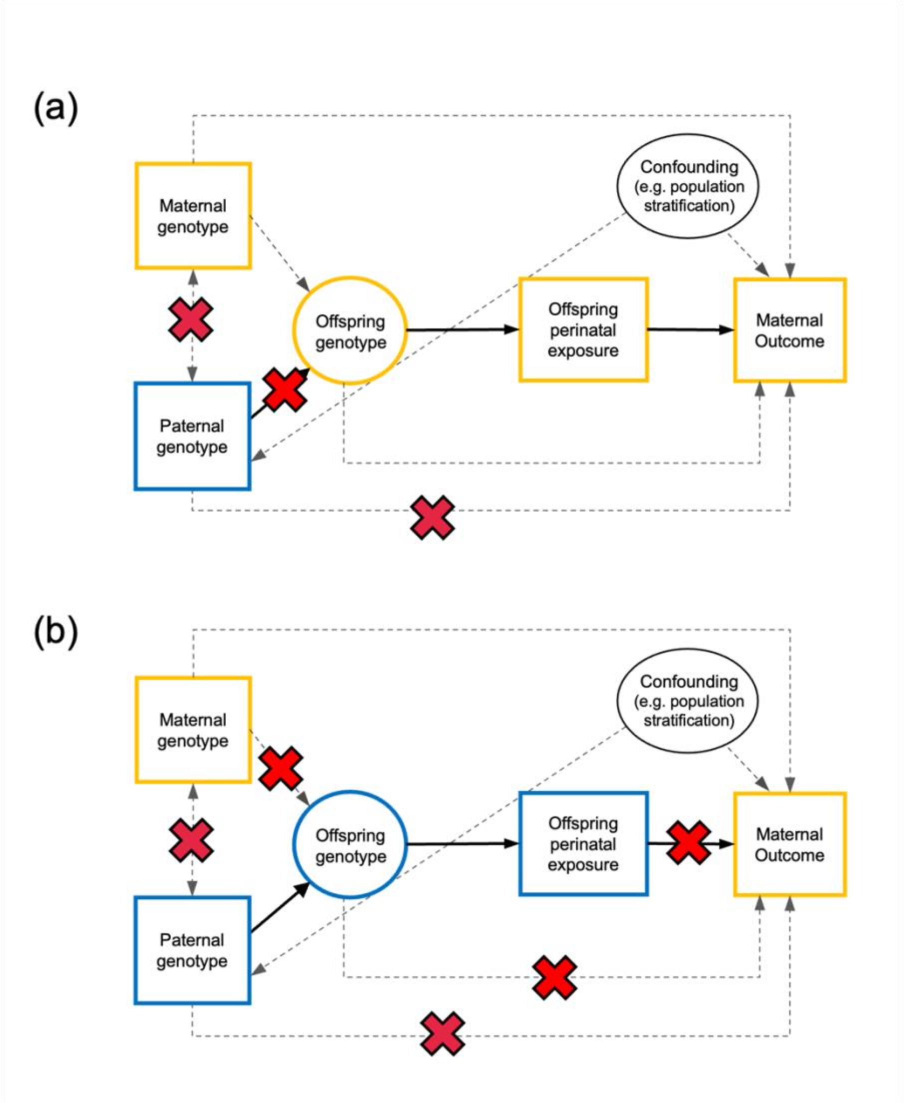

Figure S1: Causal diagrams illustrating spousal pair misclassification and its potential impact on offspring genotype by proxy Mendelian randomization (MR) analyses.
Within the offspring genotype by proxy MR model, we investigated two misclassification scenarios: (a) A genetically unrelated male is incorrectly used to proxy offspring exposure. Consequently, there is no association between “paternal” genetic variants and the offspring trait and therefore no association between paternal genetic variants and maternal outcome that is mediated through the offspring genome. This could occur when mothers report their offspring’s exposure and their own health outcome, but genetic variants from an unrelated male are incorrectly used to instrument her offspring’s exposure; (b) A genetically unrelated female is incorrectly paired with the father and his offspring. This could occur when the father reports the offspring trait, and a non-spousal female reports her own health outcome.

Supplementary Methods

Assumptions of Mendelian Randomization studies of the causal effect of offspring perinatal traits on maternal health outcomes

MR relies on a number of assumptions which have previously been discussed at length [1,2] (Figure 1a). First, MR requires that the putative genetic instruments are robustly associated with the exposure of interest in the relevant population (assumption 1 - relevance). Second, MR assumes, not unrealistically given Mendel's Laws of Segregation and Independent Assortment, that these same genetic variants are also uncorrelated with factors that affect the outcome (assumption 2 - independence). Whilst empirical studies suggest that genetic and environmental factors are likely to be independent [3], MR analyses that utilise multiple independent SNPs from different genomic regions as instrumental variables (IVs) are potentially more susceptible to violations of this assumption , in particular owing to unmodelled population structure and familial effects [4]. Third, MR assumes that the genetic variants used are only potentially associated with the outcome through the exposure of interest and not through any alternative independent pathways (assumption 3 – exclusion restriction). This last assumption is particularly problematic for the validity of MR studies and will be violated in the presence of horizontal genetic pleiotropy. This occurs when single nuclear polymorphisms (SNPs; or other genetic polymorphisms in linkage disequilibrium with them) that are associated with the exposure of interest are also associated with the outcome through pathways other than through the exposure. Given the ubiquity of pleiotropy in the human genome, this assumption is likely to be violated in many MR studies [5]. Variations of the original MR approach that are either robust to some forms of pleiotropy (E.g. MR Egger [6], weighted median, [7], and weighted mode approaches [8]) and/or permit its detection [9] have been developed.

MR also relies on the principle of gene-environment equivalence for valid causal inference. This is the notion that genetic perturbations in the exposure have the same effect on the outcome as changes in the exposure induced by environmental factors [10]. These assumptions are necessary for instrument validity and are sufficient to test for the presence of a causal effect of the exposure on the outcome. Additional assumptions are required for estimation of the magnitude of the causal effect, for example, homogeneity of the effect of the exposure on the outcome or monotonicity in the relationship between the genetic variants and the exposure. We assume that the genetic instruments act additively on the exposure of interest, with no influence of genetic dominance and epistasis. MR also relies on the assumption that genetic variants in the father are associated with the exposure of interest in the offspring during the period of interest and that this time period is relevant in terms of influencing the maternal outcome. For example, when using birth weight variants as a proxy for fetal growth, we assume these variants capture the influence of fetal growth on the mother during pregnancy.

Assumptions of offspring genotype by proxy MR

The offspring genotype by proxy MR approach involves the same three core assumptions as conventional MR analyses, however, under this approach there are additional nuances that need to be borne in mind (Figure 2). Such assumptions are discussed in detail below.

Relevance assumption

In the offspring genotype by proxy MR approach, the relevance assumption critically depends upon the accuracy of spousal matching and on the statistical strength of association between offspring genotype and offspring exposure (Figure 2a). Identification of spousal pairs in the UK Biobank has previously been inferred by matching genotyped, unrelated, opposite sex individuals on the basis of their demographic information [11-15]. However, since spousal matching has not been confirmed (e.g. by interview), this classification may be imperfect with some pairs incorrectly identified as spouses (and some spouses failing to be identified), as noted previously [13]. However, even with extensive demographic information, there is no guarantee that matching of spouses based solely on this knowledge will be perfect. In most biobank style datasets matching would be difficult to definitively confirm without interview or similar verification. It's additionally plausible that within the spousal pairs, an individual's spouse may not be the biological parent of their offspring. For example, in the Avon Longitudinal Study of Parents and Children (ALSPAC), mothers were asked whether their partner was the biological father of their child, with some participants reporting no [16], However, the availability of genotyped trios for a subset of the spousal pairs, could provide an indication of how well matching on demographic information alone is likely to perform in the larger cohort [17]. For datasets where both parents report their offspring's characteristics, this could provide further reliability. For example, in the UK biobank, both male and female participants report their number of live births/number of children fathered.

For other datasets, spousal matching may be more reliable, particularly when based on large national databases. For example, genotyped individuals in the FinnGen study can be linked to FinRegistry [18] which contains a multigenerational register includes familial relations for first-degree relatives (mother, father, children and siblings). Similarly, in the Nord-Trøndelag Health Study (the HUNT Study), information about married and cohabitating couples is provided by Statistics Norway [19]. In other cohorts, such as the Health and Retirement Study (HRS), spousal pairs were established during recruitment, where if the individual was part of a couple, their spouse or partner was also included in the sample [20].

There will of course be a proportion of spouses who do not have offspring (and therefore offspring phenotype cannot influence maternal outcome by definition), and these pairs should not be included in the main MR analysis. However, if these pairs are correctly identified, they do offer opportunities for sensitivity analyses and identification of likely IV assumption violations (see section below). Finally, confirming that the variance explained in the offspring exposure (assuming offspring phenotype is available) by the paternal genetic variant is roughly what is expected under quantitative genetics theory (i.e. one quarter of the variance of that explained by the regression of own phenotype on own genotype) can also provide an indication of the reliability of the matched data. Relevance could be compromised and/or results biased if the

parent being used to proxy offspring genotype is not the biological father of the offspring in question. The modelling implications of incorrectly matched spousal pairs (including the case where the parent being used to proxy the offspring genetic instrument is not the biological father) are discussed in the main text.

Independence assumption

The independence assumption in MR requires no confounding between the genetic variants and outcomes of interest (Figure 2b). In standard MR, indirect genetic effects, assortative mating (in previous generations) and population stratification violate this assumption and potentially lead to inconsistent estimates of the causal effect [21]. When investigating the potential causal effect of offspring traits on their mother's health using MR, another potential source of confounding is the maternal genome. This is because maternal genotype will be correlated with offspring genotype (through transmission) and may also plausibly influence the maternal outcome. In the offspring genotype by proxy MR approach, we utilise the paternal genotype to proxy offspring genotype which should be uncorrelated with maternal genotype in the absence of assortative mating and therefore protect MR analyses from confounding by the maternal genome. This relies on the assumption of random mating for the exposure.

There is evidence that spouses typically exhibit greater similarity than would be expected by chance for a variety of traits including height and BMI. It appears that in many cases this increased similarity is due to phenotypic assortment rather than social homogamy or convergence in phenotypes over time [12]. Assortative mating between spousal pairs induces a correlation between spousal genotypes at loci on which the assortment is based (path 2b in Figure 2b). Thus, phenotypic assortment has the potential to bias causal estimates in the offspring genotype by proxy MR design if the trait undergoing assortment is the exposure of interest or is genetically correlated with (or causes) the exposure. Such assortment can induce correlation between the genetic instruments (or variants in LD with them) in the parents. This potentially opens a path between genetic variants (or variants in LD with them) in the father and the outcome in the mother if the genetic variants for the exposure in the mother are associated with the outcome (Figure 2b path 3b). We argue that assortment on the perinatal trait is extremely unlikely, however, recognise that perinatal exposures may be indirectly influenced by parental phenotypes where assortment is more likely. The phenotypic correlation between spouses for the exposure in the parental generation should provide an indication of the degree to which assortment is likely to be a concern and where caution is warranted. However, we note that for these phenotypes, assortment is problematic not only for our framework, but for most population-based MR methods more broadly [21].

Exclusion restriction assumption

The exclusion restriction assumption stipulates the genetic instrument must only be associated with the outcome through the exposure. In the current context, it is therefore assumed that there is no directed path between paternal genetic variants and maternal outcome other than that going through the offspring exposure (Figure 2c). In traditional MR analyses, the exclusion restriction may be violated by horizontal genetic pleiotropy, a feature of the human genome that is likely to be common if not endemic [5]. This is also the case in offspring genotype by proxy MR

where pleiotropic paths from offspring genotype to maternal outcome may also invalidate the use of the paternal genetic variants as instruments (Figure 2c path 1c). However, the offspring genotype by proxy MR framework also has an additional complication, namely the possibility that paternal genetic variants may affect the maternal outcome through the paternal phenotype (Figure 2c path 2c). For example, this assumption would be violated if the exposure of interest, when present in the father were causal for the maternal outcome. For perinatal traits, it is reasonable to assume no causal impact of paternal phenotype on the maternal outcome for some exposures (e.g. if the interest were on the effect of offspring birthweight on maternal health, it is unlikely that paternal birthweight could affect maternal health).

Another potential violation of the exclusion restriction assumption is if the paternal genetic variants are pleiotropic for other paternal phenotypes (that are not the proxied exposure) that in turn affect the maternal outcome (Figure 2c path 2c). Potential violations of this assumption could be investigated by examining whether the genetic variants are associated with other paternal phenotypes likely to affect the maternal outcome, as well as employing MR sensitivity analyses within the offspring genotype by proxy MR context. Where violations are identified, it may be possible to perform sub-analyses excluding the potentially pleiotropic variants. Similarly, the presence of horizontal pleiotropy can be investigated by incorporating gene by environment interactions into the offspring genotype by proxy MR approach as discussed in the main text [22,23]. While dynastic effects of paternal genotype on offspring exposure (Figure 2c path 3c) do not inherently violate the exclusion restriction assumption, these must be accounted for when estimating the causal effect.

Additional considerations – Indirect and parent of origin paternal genetic effects on the offspring exposure

The offspring genotype by proxy MR approach, leverages the assumption that the genetic variants for the exposure in the father exert their effect on the offspring exposure through the offspring's genome (i.e. following transmission of alleles from father to offspring). However, these same paternal genotypes may also exert indirect (or dynastic) effects [24,25] or parent of origin (PofO) effects on offspring exposure. Provided the paternal genetic variants are (strongly) marginally associated with the offspring exposure (i.e. direct offspring and indirect paternal genetic effects on the offspring exposure don't cancel out), this fact should not be problematic in and of itself for causal inference, as both paternal and offspring genetic variants may constitute valid IVs. We show mathematically that dynastic and PofO effects of the paternal genotype on the offspring exposure does not result in inconsistent estimates of the causal effect in the offspring genotype by proxy MR design in the supplementary material below. Of note, paternal genetic variants that exert indirect or PofO effects on some offspring exposures may also be more likely to be correlated with or exert effects on other offspring phenotypes and/or maternal phenotypes (see discussion of the independence and exclusion restriction assumptions above). For example, paternal effects that promote high birth weight (e.g. nutrition and food choices) may associated with paternal BMI that could conceivably affect a range of maternal phenotypes through pathways other than through offspring birth weight. For this reason, we recommend using genetic variants that have a direct genetic effect on the exposure (i.e. do not exhibit maternal or paternal genetic effects).

Inconsistency of instrumental variable estimators under pleiotropy

The large sample properties of each instrumental variable estimator can be derived under asymptotic theory. For the subsequent derivations, we define the following quantities:

Z_p = the genetic variant in the fathers

Z_m = the genetic variant in the mothers

Z_o = the genetic variant in the offspring

Z_{pt} = the allele transmitted from father to offspring

X = offspring exposure

Y = maternal outcome

$\beta_{ZO,X}$ = population level association between offspring genetic variant and offspring exposure

β_{XY} = population level causal effect of offspring exposure on maternal outcome

$\beta_{ZO,Y}$ = population level effect of offspring genetic variant on maternal outcome

$\beta_{ZP,Y}$ = population level effect of paternal genetic variant on maternal outcome

$\beta_{ZP,X}$ = population level effect of paternal genetic variant on offspring exposure

For all derivations, we assume absence of assortative mating and genetic confounding through the maternal genome.

In the case of the offspring genotype by proxy MR design, the causal effect in large samples is given by:

$$\text{plim}(\hat{\beta}_{IV}) = \text{plim}\left(\frac{\text{cov}(Z_P,Y)}{\text{cov}(Z_P,X)}\right) = \frac{\text{COV}(Z_P,Y)}{\text{COV}(Z_P,X)} = \frac{(\beta_{ZP,Y}+0.5\times\beta_{ZO,Y}+0.5\times\beta_{XY}\times\beta_{ZO,X})\times\text{var}(Z_P)}{0.5\times\beta_{ZO,X}\times\text{var}(Z_P)} = \beta_{XY} + \frac{\beta_{ZO,Y}}{\beta_{ZO,X}} + \frac{2\beta_{ZP,Y}}{\beta_{ZO,X}}$$

where plim is the probability limit, cov refers to the sample covariance, and COV and VAR the covariance and variance in the population respectively.

In the case of MR with adjustment for maternal genotype, the causal effect in large samples is given by:

$$\text{plim}(\hat{\beta}_{IV}) = \text{plim}\left(\frac{\text{cov}(Z_O,Y)}{\text{cov}(Z_O,X)}\right) = \frac{\text{COV}(Z_O,Y)}{\text{COV}(Z_O,X)} = \frac{\beta_{XY}\times\beta_{ZO,X}\times\text{var}(Z_O)+0.5\times\beta_{ZP,Y}\times\text{var}(Z_P)+\beta_{ZO,Y}\times\text{var}(Z_O)}{\beta_{ZO,X}\times\text{var}(Z_O)} = \beta_{XY} + \frac{\beta_{ZO,Y}}{\beta_{ZO,X}} + \frac{\beta_{ZP,Y}}{2\beta_{ZO,X}}$$

In the case of MR using paternally transmitted alleles, the causal effect in large samples is given by:

$$\text{plim}(\hat{\beta}_{IV}) = \text{plim}\left(\frac{\text{cov}(Z_{Pt},Y)}{\text{cov}(Z_{Pt},X)}\right) = \frac{\text{COV}(Z_{Pt},Y)}{\text{COV}(Z_{Pt},X)}$$

$$= \frac{\beta_{XY}\times\beta_{ZO,X}\times 0.5\times\text{var}(Z_O)+\beta_{ZO,Y}\times 0.5\times\text{var}(Z_O)+\beta_{ZP,Y}\times 0.5\times\text{var}(Z_O)}{\beta_{ZO,X}\times 0.5\times\text{var}(Z_O)}$$

$$= \beta_{XY} + \frac{\beta_{ZO,Y}}{\beta_{ZO,X}} + \frac{\beta_{ZP,Y}}{\beta_{ZO,X}}$$

For the last derivation, we assume that the parental origins of the alleles transmitted to the offspring can be determined with certainty.

Finally, we show that direct effects of the paternal genotype on the offspring exposure (either through dynastic or parent of origin effects) does not result in inconsistent estimates of the causal effect in the offspring genotype by proxy MR design:

$$\mathrm{plim}(\hat{\beta}_{IV}) = \mathrm{plim}\left(\frac{\mathrm{cov}(Z_P, Y)}{\mathrm{cov}(Z_P, X)}\right) = \frac{\mathrm{COV}(Z_P, Y)}{\mathrm{COV}(Z_P, X)}$$
$$= \frac{0.5 \times \beta_{XY} \times \beta_{ZO,X} \times \mathrm{var}(Z_P) + \beta_{XY} \times \beta_{ZP,X} \times \mathrm{var}(Z_P)}{0.5 \times \beta_{ZO,X} \times \mathrm{var}(Z_P) + \beta_{ZP,X} \times \mathrm{var}(Z_P)} = \beta_{XY}$$

Methods for data simulations

In this section we describe the methods for data simulation presented in this manuscript following the ADEMP guidelines [26]. We first describe the data generating mechanism common across all simulations.

Common gata-generating mechanism

Maternal, paternal and offspring genotypes were at a single genetic locus (even if not all of these were used in the analysis). Genotypes were simulated assuming a trait increasing allele frequency of q = 0.6 and standard autosomal Mendelian inheritance by sampling from a Binomial distribution. Standardized additive genotypic dosages (mean zero and unit variance) for maternal (Z_m), paternal (Z_p) and offspring (Z_o) genotypes were calculated. For each family *i,* the offspring exposure X was generated using the following equation:

$$X_i = \sqrt{V_q} \times Z_{o_i} + \beta_{UX} \times U_i + \delta_i$$

where V_q denotes the variance in the offspring exposure explained by offspring genotype, Z_o is a latent variable of unit variance indexing the offspring genotype, U is a standard normal random variable representing (unmeasured) confounding influences, β_{UX} denotes the total effect of latent confounders U on the offspring exposure X, and δ is a normally distributed random variable with mean zero. The variance of δ is such that X has unit variance.

The maternal outcome Y (for each family *i*) was generated according to the following model:

$$Y_i = \beta_{XY} \times X_i + \beta_{UY} \times U_i + \sqrt{V_o} \times Z_{o_i} + \sqrt{V_p} \times Z_{p_i} + \sqrt{V_m} \times Z_{m_i} + \epsilon_i$$

where β_{XY} is the causal effect of the offspring exposure X on the maternal outcome Y, β_{UY} is the total effect of confounding variables on the maternal outcome, V_o denotes the variance in the maternal outcome explained by offspring genotype, V_p denotes the variance in the maternal outcome explained by paternal genotype, Z_p is a latent variable of unit variance indexing the paternal genetic instrument, V_m denotes the variance in the maternal outcome explained by maternal genotype, Z_m is a latent variable of unit variance indexing the maternal genetic instrument, and ϵ is a random normal variate with mean zero with variance such that Y has unit variance asymptotically.

β_{XY} represents the causal effect of the offspring exposure X on the maternal outcome Y that we estimate from the MR analyses. However, we know (as shown in the Figure 1d) that this will likely be biased because in the presence of non-zero paths from the offspring genetic instrument Z_o and the paternal genetic instrument Z_p to the maternal outcome Y. Unless specified, we do not consider the potential effects of pleiotropy (i.e. variance in the maternal

outcome explained by offspring (V_o), maternal (V_m) and paternal (V_p) genotype is set to zero) or assortative mating (Z_p and Z_m are independently simulated).

(1) Horizontal pleiotropy and weak instruments

Aims: Estimate the magnitude and direction of bias in the causal effect estimate under realistic settings of horizontal pleiotropy and weak instruments.

Data-generating mechanism: Data were generated following the mechanisms outlined above for 50,000 parent offspring trios. We considered the following conditions: (1) variance in the maternal outcome explained by offspring (V_o), maternal (V_m) and paternal (V_p) genotype (i.e. horizontal pleiotropy or confounding; each assessed at three conditions of 0, 1% or 5%), (2) variance in the offspring exposure explained by offspring genotype (two conditions corresponding to strong or weak instruments: V_q = 2% or 0.5%), (3) the strength of the causal relationship between offspring exposure and maternal outcome (two conditions: β_{XY} = 0.2 or 0), (4) the strength of confounding between the offspring exposure and maternal outcome (β_{UX} = β_{UY} =0.1), and selected informative combinations of these factors.

Estimand: The causal effect of the offspring exposure on the maternal outcome.

Method: MR analyses were performed using two-stage least squares using the ivreg R package version 0.6-5 [27]. We compared the three MR approaches in Table 1 for estimating the causal effect of offspring exposure on maternal outcome.

Performance measures: For each scenario, we generated 1,000 replicates and recorded the instrumental variables estimate of the causal relationship and its standard error.

(2) The magnitude of bias in the causal effect estimates and power to detect a causal effect under spousal pair misclassification

Aim: Estimate the impact of (accidental) inclusion of incorrectly matched spousal pairs (or correctly matched spousal pairs that are not biological parents) on bias in the causal effect estimate and statistical power. We consider two misclassification scenarios: (1) A male who is genetically unrelated to the offspring under consideration is incorrectly used to the proxy offspring exposure. Consequently, there is no association between paternal genetic variants, offspring genotype and offspring exposure and therefore no association between paternal genetic variants and maternal outcome through this pathway. This could occur when mothers report their offspring's exposure and their own health outcome, but genetic variants from an unrelated male are incorrectly used to instrument her offspring's exposure; (2) A genetically unrelated female is incorrectly paired with the father and his offspring. This could occur when the father reports the offspring trait, and a non-spousal female reports her own health outcome.

Data-generating mechanism: Data were generated following the mechanisms outlined above for 50,000 parent offspring trios. We generated misclassified spousal pairs as described below. For scenario 1, a male who is genetically unrelated to the offspring and independent of the maternal outcome under consideration. The genetic variant from the unrelated male was then used to the proxy offspring exposure. For scenario 2, we simulated a maternal outcome that is independent of the paternal genotype and offspring exposure under consideration.

Estimand (target): The causal effect of the offspring exposure on the maternal outcome and the null hypothesis i.e. power to detect a true causal effect.

The following conditions were considered: (1) varying the proportion of randomly misclassified spouses (0%, 10%, 20%, 30% and 40% of spousal pairs), (2) variance in the offspring exposure explained by offspring genotype (two conditions: V_q = 0.5% or 2%), (3) the strength of the causal relationship between offspring exposure and maternal outcome (two conditions: β_{XY} = 0.2 or 0), (4) the strength of confounding between the offspring exposure and maternal outcome (β_{UX} = β_{UY} = 0.1), and select informative combinations of these factors.
Method: MR analyses for the offspring genotype by proxy approach were performed using two-stage least squares using the ivreg R package version 0.6-5 [27].
Performance measures: For each scenario, we generated 1,000 replicates and recorded the instrumental variables estimate of the causal relationship and its standard error. We additionally calculate the power to detect a causal effect (defined as the proportion of simulations that reject the null hypothesis assuming a 5% type I error rate), coverage (defined as the proportion of simulations where the true causal effect lies within the 95% confidence interval for the estimand) and the F-statistic of the association between genetic instrument and offspring exposure.

(3) The power to detect a causal effect of an offspring exposure on a maternal health outcome using MR

Aims: Compare estimates of the power to detect a causal effect of an offspring exposure on a maternal health outcome using three MR approaches
Data-generating mechanism: Data were generated following the mechanisms outlined above for parent offspring trios. For each of the MR approaches considered we use sample size to refer to the relevant observational unit as per the study design i.e. a spousal pair, a mother-offspring pair, a parent offspring trio. The following conditions were considered: (1) sample sizes ranging from 7,000 to 300,000, (2) variance in the offspring exposure explained by offspring genotype (ranging from: V_q = 1% to 10%, (3) the strength of confounding between the offspring exposure and maternal outcome (β_{UX} = β_{UY} = 0.1) and (4) the strength of the causal relationship between offspring exposure and maternal outcome (two conditions: β_{XY} = 0.1).
Estimand (target): Null hypothesis (i.e. power to detect a true causal effect).
Method: MR analyses were performed using two-stage least squares using the ivreg R package version 0.6-5 [27]. We compared the three MR approaches in Table 1 for estimating the causal effect of offspring exposure on maternal outcome.
Performance measures: For each scenario, we generated 1,000 replicates and recorded the instrumental variables estimate of the causal relationship and its standard error. We calculated the power to detect a causal effect (defined as the proportion of simulations that reject the null hypothesis assuming a 5% type I error rate).

References

1. Lawlor D, Richmond R, Warrington N, et al. Using Mendelian randomization to determine causal effects of maternal pregnancy (intrauterine) exposures on offspring outcomes: Sources of bias and methods for assessing them. *Wellcome Open Res*. Feb 14 2017;2:11. doi:10.12688/wellcomeopenres.10567.1
2. Evans DM, Davey Smith G. Mendelian Randomization: New Applications in the Coming Age of Hypothesis-Free Causality. *Annu Rev Genomics Hum Genet*. 2015;16:327-50. doi:10.1146/annurev-genom-090314-050016
3. Smith GD, Lawlor DA, Harbord R, Timpson N, Day I, Ebrahim S. Clustered environments and randomized genes: a fundamental distinction between conventional and genetic epidemiology. *PLoS Med*. Dec 2007;4(12):e352. doi:10.1371/journal.pmed.0040352
4. Brumpton B, Sanderson E, Heilbron K, et al. Avoiding dynastic, assortative mating, and population stratification biases in Mendelian randomization through within-family analyses. *Nature Communications*. 2020/07/14 2020;11(1):3519. doi:10.1038/s41467-020-17117-4
5. Evans DM, Brion MJA, Paternoster L, et al. Mining the Human Phenome Using Allelic Scores That Index Biological Intermediates. *PLOS Genetics*. 2013;9(10):e1003919. doi:10.1371/journal.pgen.1003919
6. Bowden J, Davey Smith G, Burgess S. Mendelian randomization with invalid instruments: effect estimation and bias detection through Egger regression. *International Journal of Epidemiology*. 2015;44(2):512-525. doi:10.1093/ije/dyv080
7. Bowden J, Davey Smith G, Haycock PC, Burgess S. Consistent Estimation in Mendelian Randomization with Some Invalid Instruments Using a Weighted Median Estimator. *Genetic Epidemiology*. 2016;40(4):304-314. doi:10.1002/gepi.21965
8. Hartwig FP, Davey Smith G, Bowden J. Robust inference in summary data Mendelian randomization via the zero modal pleiotropy assumption. *International Journal of Epidemiology*. 2017;46(6):1985-1998. doi:10.1093/ije/dyx102
9. Bowden J, Hemani G, Davey Smith G. Invited Commentary: Detecting Individual and Global Horizontal Pleiotropy in Mendelian Randomization—A Job for the Humble Heterogeneity Statistic? *American Journal of Epidemiology*. 2018;187(12):2681-2685. doi:10.1093/aje/kwy185
10. Sanderson E, Glymour MM, Holmes MV, et al. Mendelian randomization. *Nat Rev Methods Primers*. Feb 10 2022;2doi:10.1038/s43586-021-00092-5
11. Tenesa A, Rawlik K, Navarro P, Canela-Xandri O. Genetic determination of height-mediated mate choice. *Genome Biol*. 2016/01/19 2016;16(1):269. doi:10.1186/s13059-015-0833-8
12. Robinson MR, Kleinman A, Graff M, et al. Genetic evidence of assortative mating in humans. *Nature Human Behaviour*. 2017/01/09 2017;1(1):0016. doi:10.1038/s41562-016-0016
13. Howe LJ, Lawson DJ, Davies NM, et al. Genetic evidence for assortative mating on alcohol consumption in the UK Biobank. *Nature Communications*. 2019/11/19 2019;10(1):5039. doi:10.1038/s41467-019-12424-x
14. Yengo L, Robinson MR, Keller MC, et al. Imprint of assortative mating on the human genome. *Nature Human Behaviour*. Dec 2018;2(12):948-954. doi:10.1038/s41562-018-0476-3
15. Sjaarda J, Kutalik Z. Partner choice, confounding and trait convergence all contribute to phenotypic partner similarity. *Nat Hum Behav*. May 2023;7(5):776-789. doi:10.1038/s41562-022-01500-w

16. Golding J, Bickerstaffe I, Iles-Caven Y, Northstone K. Paternal health in the first 12-13 years of the ALSPAC study. *Wellcome Open Res*. 2023;8:8. doi:10.12688/wellcomeopenres.18639.1
17. Hatton AA, Brito Nunes C, Flatley C, Lawlor DA, Evans DM. Utilising novel Mendelian randomization approaches to investigate a potential causal effect of fetal growth on maternal cardiometabolic risk in the UK Biobank. *medRxiv*. 2025:2025.07.17.25331747. doi:10.1101/2025.07.17.25331747
18. Viippola E, Kuitunen S, Rodosthenous RS, et al. Data Resource Profile: Nationwide registry data for high-throughput epidemiology and machine learning (FinRegistry). *International Journal of Epidemiology*. 2023;52(4):e195-e200. doi:10.1093/ije/dyad091
19. Bjørngaard JH, Vie GÅ, Krokstad S, Janszky I, Romundstad PR, Vatten LJ. Cardiovascular mortality – Comparing risk factor associations within couples and in the total population – The HUNT Study. *International Journal of Cardiology*. 2017/04/01/ 2017;232:127-133. doi:10.1016/j.ijcard.2017.01.041
20. Sonnega A, Faul JD, Ofstedal MB, Langa KM, Phillips JW, Weir DR. Cohort Profile: the Health and Retirement Study (HRS). *Int J Epidemiol*. Apr 2014;43(2):576-85. doi:10.1093/ije/dyu067
21. Hartwig FP, Davies NM, Davey Smith G. Bias in Mendelian randomization due to assortative mating. *Genet Epidemiol*. Oct 2018;42(7):608-620. doi:10.1002/gepi.22138
22. Davey Smith G. Use of genetic markers and gene-diet interactions for interrogating population-level causal influences of diet on health. *Genes Nutr*. Feb 2011;6(1):27-43. doi:10.1007/s12263-010-0181-y
23. Spiller W, Slichter D, Bowden J, Davey Smith G. Detecting and correcting for bias in Mendelian randomization analyses using Gene-by-Environment interactions. *Int J Epidemiol*. Jun 1 2019;48(3):702-712. doi:10.1093/ije/dyy204
24. Kong A, Thorleifsson G, Frigge ML, et al. The nature of nurture: Effects of parental genotypes. *Science*. 2018;359(6374):424-428. doi:doi:10.1126/science.aan6877
25. Warrington NM, Freathy RM, Neale MC, Evans DM. Using structural equation modelling to jointly estimate maternal and fetal effects on birthweight in the UK Biobank. *Int J Epidemiol*. Aug 1 2018;47(4):1229-1241. doi:10.1093/ije/dyy015
26. Morris TP, White IR, Crowther MJ. Using simulation studies to evaluate statistical methods. *Statistics in Medicine*. 2019;38(11):2074-2102. doi:10.1002/sim.8086
27. *ivreg: Instrumental-Variables Regression by '2SLS', '2SM', or '2SMM', with Diagnostics*. Version 0.6-3. 2024.
